# Alcohol insensitivity and the incentive salience of alcohol: Two decades of work relevant to future directions of the addictions neuroclinical assessment

**DOI:** 10.1038/s41398-025-03273-y

**Published:** 2025-02-14

**Authors:** Roberto U. Cofresí, Thomas M. Piasecki, Bruce D. Bartholow

**Affiliations:** 1 Department of Psychological Sciences, University of Missouri – Columbia, Columbia, MO USA; 2 Department of Medicine and Center for Tobacco Research and Intervention, University of Wisconsin-Madison, Madison, WI USA; 3 Department of Psychological and Brain Sciences, University of Iowa, Iowa City, IA USA

**Keywords:** Addiction, Human behaviour, Neuroscience, Biomarkers

In their recent article in this journal, Gunawan and colleagues report evidence that the Incentive Salience (IS) domain within the proposed Addictions Neuroclinical Assessment (ANA) framework is represented by variance in alcohol motivation and alcohol insensitivity [1]. This finding accords with nearly two decades of research from our labs suggesting a link between alcohol insensitivity and the IS of alcohol-related cues. In the interest of accelerating discovery in this area, here we provide a selective review of this work.

The attribution of IS to a reward-predictive cue transforms the cue into a “motivational magnet” [2] that: (i) captures attention; (ii) impels approach; (iii) serves as a conditioned reward; (iv) can invigorate reward-seeking actions; and (v) can induce a bio-behavioral state of motivation for the anticipated reward (i.e., craving). Our research has demonstrated that nearly all these manifestations of IS are amplified among individuals reporting lower sensitivity (LS) to acute alcohol (viz., alcohol insensitivity) relative to their higher-sensitivity (HS) peers.

Using data from behavioral and event-related brain potential (ERP) measures, these studies indicate that naturally learned visual cues for alcohol (e.g., pictures of alcohol beverages) capture and hold visuospatial attention more strongly among LS versus HS individuals [3], an effect attributable to the cues’ enhanced affective-motivational significance among LS individuals [4, 5]. Such cues also activate approach behavior among LS (but not HS) individuals [5, 6]. Additionally, naturally learned olfactory cues for alcohol are more potent conditioned rewards for individuals with the LS relative to HS phenotype [7].

These various laboratory observations have analogues in measurements recorded in LS individuals’ natural drinking environments. Ecological momentary assessment studies have shown that, relative to their HS counterparts, individuals with LS phenotypes exhibit greater alcohol cue-provoked subjective craving in natural drinking contexts [8, 9].

Most of our studies have tested individuals ages 18–20, for whom laboratory alcohol administration is illegal in the U.S. In such samples, we make LS/HS classifications based on responses to a retrospective self-report scale querying numbers of drinks required to experience each of 15 effects from drinking alcohol [10]. This scale has been validated using placebo-controlled alcohol administration in the laboratory (with individuals ages 21 + ) and correlates strongly [10] with scores from the alcohol insensitivity scale [11] used by Gunawan and colleagues. This empirical overlap adds to the conceptual overlap between the work reviewed in this article and the findings reported by Gunawan and colleagues.

In their article, Gunawan and colleagues call for inquiry into the neurobiological correlates of the different ANA domains and factors, including the link between alcohol insensitivity and IS. We echoed the latter in our recent report about the neurobiological correlates of differential reactivity to visual alcohol cues among individuals varying in alcohol sensitivity [12]. Using functional magnetic resonance imaging (fMRI), this pilot study showed enhanced responses to visual cues for alcohol in the putamen and prefrontal and orbitofrontal cortices among LS compared to HS individuals, especially among those using alcohol at hazardous levels. Amplified responses in these nodes of the mesocorticolimbic system are consistent with the possibility that LS individuals are at elevated risk for sensitization of the mesocorticolimbic system to alcohol and its cues. fMRI studies in larger samples, and especially prospective studies across periods of elevated risk for AUD onset/progression, are needed to confirm this possibility and to determine the psychometric reliability and prognostic utility of fMRI-derived measures.

As emphasized by Gunawan and colleagues, it is critical to consider measures other than self-report in the continued development of the ANA battery. The IS construct as developed in preclinical models has proven difficult to translate to a human model, particularly because it is not synonymous with the phenomenon of self-reported craving for alcohol in humans. To advance development of a human analogue of IS, researchers must identify reliable measures of the IS construct (and its correlates, including alcohol insensitivity) at multiple units of analysis (e.g., self-report, behavioral, and neuro-physiological) from which multimodal latent factors representing variance shared across these units might be constructed. The clinical utility of such an approach is well documented [13]. Critically, for this approach to succeed, measures at each unit of analysis must be both valid and reliable. Unfortunately, IS measurement paradigms often rely on behavioral tasks and/or fMRI measures that were optimized to highlight ubiquitous within-person effects rather than to characterize stable between-person differences, limiting their utility for individual-differences research. Like Gunawan et al., we believe careful attention to psychometrics in neuroclinical assessment is crucial. Without robustly reliable assessment, compelling frameworks like the ANA cannot move beyond the heuristic to the clinically utilitarian.

Better understanding of the IS construct in humans, and particularly its association with alcohol insensitivity, also critically depends on the development of strong theoretical models. We have argued that alcohol insensitivity may confer risk for AUD, in part, via susceptibility to IS sensitization [14]. Briefly, alcohol insensitivity may be a trait-like marker of heritable innate variations in the mesocorticolimbic system that predispose a person to imbue alcohol-predictive cues with more IS across repeated experiences of alcohol use-related rewards, such that cues become progressively more powerful drivers of the alcohol use cycle. Alternatively, alcohol insensitivity may be acquired rather than innate, and since LS individuals drink more than their HS peers, IS sensitization may reflect differential accrual of functional adaptations in the mesocorticolimbic system due chronic intermittent exposure of the brain to higher concentrations of alcohol in LS compared to HS individuals. We routinely observe that differences in alcohol cue IS between individuals with LS versus HS phenotypes are robust to covarying differences in typical alcohol use, suggesting that alcohol insensitivity-related risk for AUD is not fully explained by differential chronic exposure. However, disentangling the role of acquired vs. innate alcohol insensitivity in susceptibility to IS sensitization necessitates longitudinal studies that span developmental stages and assess constructs earlier in the lifespan.

Our focus on heightened motivational reactivity to alcohol cues as a manifestation of IS sensitization is grounded in theory [2]. However, the same theory also suggests that IS sensitization may manifest as heightened sensitivity to the stimulating effects of alcohol consumption [15]. In prior work, we have reported that individuals classified as LS based on retrospective self-report experience greater stimulation and lesser sedation after alcohol consumption in the lab, relative to their HS peers [10]. To advance understanding of how alcohol sensitivity phenotypes might contribute to the development of AUD *via* IS sensitization, future studies—especially longitudinal studies—should incorporate multi-domain measurement of IS with placebo-controlled measures of stimulation and sedation from alcohol.

In sum, continued work on the IS domain of the ANA can be expected to advance understanding of both the contributions of alcohol insensitivity to alcohol IS and the processes by which LS confers risk for AUD.
